# p120-catenin subfamily members have distinct as well as shared effects on dendrite morphology during neuron development *in vitro*

**DOI:** 10.3389/fncel.2023.1151249

**Published:** 2023-04-04

**Authors:** Maxsam S. Donta, Yogesh Srivastava, Christina M. Di Mauro, Adriana Paulucci-Holthauzen, M. Neal Waxham, Pierre D. McCrea

**Affiliations:** ^1^Department of Genetics, University of Texas MD Anderson Cancer Center, Houston, TX, United States; ^2^Program in Genetics and Epigenetics, Graduate School of Biomedical Sciences, The University of Texas Health Science Center at Houston, Houston, TX, United States; ^3^Department of Neurobiology and Anatomy, McGovern Medical School, The University of Texas Health Science Center at Houston, Houston, TX, United States; ^4^Program in Neuroscience, Graduate School of Biomedical Sciences, The University of Texas Health Science Center at Houston, Houston, TX, United States

**Keywords:** catenin, dendrite morphogenesis, dendrite branching, p120-catenin subfamily, neuron morphology

## Abstract

Dendritic arborization is essential for proper neuronal connectivity and function. Conversely, abnormal dendrite morphology is associated with several neurological pathologies like Alzheimer’s disease and schizophrenia. Among major intrinsic mechanisms that determine the extent of the dendritic arbor is cytoskeletal remodeling. Here, we characterize and compare the impact of the four proteins involved in cytoskeletal remodeling–vertebrate members of the p120-catenin subfamily–on neuronal dendrite morphology. In relation to each of their own distributions, we find that p120-catenin and delta-catenin are expressed at relatively higher proportions in growth cones compared to ARVCF-catenin and p0071-catenin; ARVCF-catenin is expressed at relatively high proportions in the nucleus; and all catenins are expressed in dendritic processes and the soma. Through altering the expression of each p120-subfamily catenin in neurons, we find that exogenous expression of either p120-catenin or delta-catenin correlates with increased dendritic length and branching, whereas their respective depletion decreases dendritic length and branching. While increasing ARVCF-catenin expression also increases dendritic length and branching, decreasing expression has no grossly observable morphological effect. Finally, increasing p0071-catenin expression increases dendritic branching, but not length, while decreasing expression decreases dendritic length and branching. These distinct localization patterns and morphological effects during neuron development suggest that these catenins have both shared and distinct roles in the context of dendrite morphogenesis.

## Introduction

Dendrites were discovered over 100 years ago, and perhaps their most notable feature is their elaborate range of morphologies that make up the dendritic arbor. Through precise extension, branching, elongation, retraction, and pruning, dendritic arbors allow neurons to connect with each other to form neural circuits (Torben-Nielsen and Cuntz, 2014). Conversely, abnormal dendrite morphology is seen in neurological diseases such as Alzheimer’s disease, schizophrenia, and Down Syndrome (Kaufmann and Moser, 2000; Elston, 2003; Kolomeets et al., 2007; Couch et al., 2010; Kulkarni and Firestein, 2012; Martinez-Cerdeño, 2017). While there is much to be discovered about the mechanisms of dendritic arborization, there are several known molecular mechanisms of control (Jan and Jan, 2010; de la Torre-Ubieta and Bonni, 2011; Arikkath, 2012). These mechanisms are carried out through different categories of proteins, which include cell adhesion molecules, cytoskeleton regulators, cell surface receptors, secreted molecules, postsynaptic density proteins, secreted molecules, signaling molecules, transcription factors, calcium signaling proteins, and molecules that control Golgi trafficking (Yacoubian and Lo, 2000; Redmond et al., 2002; Huang et al., 2005; Charych et al., 2006; Elia et al., 2006; Ye et al., 2007; Puram et al., 2011; Arikkath, 2012). Of these various classes, our study focuses on catenins, which are involved in cell adhesion and cytoskeletal reorganization. More specifically, this study investigates and compares how each member in the p120-catenin subfamily affects dendrite morphogenesis during neuron development.

**FIGURE 1:**
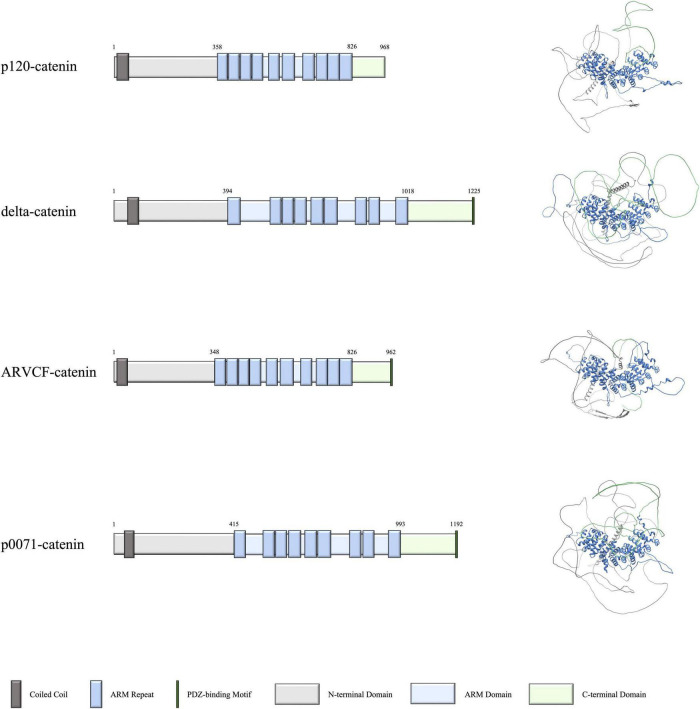
Domains and structures of human p120-catenin subfamily proteins. Schematics on the left depict the domains of p120-catenin, delta-catenin, ARVCF-catenin, and p0071-catenin, with the corresponding predicted structure of each protein on the right. Data for domain schematics was obtained from UniProt, and models were made using AlphaFold (The UniProt Consortium, 2018; Jumper et al., 2021).

The vertebrate p120-catenin subfamily is made up of four proteins: p120-catenin (gene name CTNND1), delta-catenin (gene name CTNND2), ARVCF-catenin (gene name ARVCF), and p0071-catenin (gene name PKP4). This subfamily of proteins is characterized by a central series of 9 or 10 Armadillo (ARM) repeats that are located between each catenin’s N- and C-terminal tails. Of these four proteins, all except p120-catenin contains a PDZ-binding motif at the C-terminus, which enables them to selectively bind to partner proteins that contain appropriate PDZ domains (Yuan and Arikkath, 2017). The domains and structures of each catenin are shown in Figure 1. Starting from the N-terminus, the coiled coil domain is thought to recruit other catenin binding partners to the cadherin-catenin complex (Markham et al., 2014). The ARM repeats in the middle of the catenins facilitate a variety of associations. Perhaps most notably, certain ARM repeats enable the binding of catenins to classical cadherins and are one of the sites of interactions with small Rho GTPases (Yanagisawa et al., 2008). Such interactions help the p120-subfamily of catenins to localize properly and to modulate the cytoskeleton, as well as enable additional functions such as stabilizing cadherin-catenin complexes at adherens junctions, participating in intracellular transport, and regulating cell polarity (Anastasiadis and Reynolds, 2000; Laura et al., 2002; Hatzfeld, 2005; McCrea and Park, 2007; Baumert et al., 2020).

While p120-catenin, ARVCF-catenin, and p0071-catenin are widely distributed throughout mammalian tissues, including the central nervous system, delta-catenin expression is primarily restricted to the central nervous system (Hatzfeld and Nachtsheim, 1996; Paffenholz and Franke, 1997; Sirotkin et al., 1997; Keirsebilck et al., 1998; Kawamura et al., 1999; Paulson et al., 2000; Duparc et al., 2006; Munton et al., 2007; Huttlin et al., 2010; Erickson et al., 2015; Foldy et al., 2016; Lu et al., 2016). Furthermore, p120-catenin and delta-catenin are also widely expressed at the protein level in the mammalian brain during early development. While studies have not yet shown the relative extent to which ARVCF-catenin and p0071-catenin are present at the protein level in early development, they are each present at the RNA level (Chauvet et al., 2003; Yuan and Arikkath, 2017). Within neurons, p120-catenin has been shown to localize to growth cones as well as presynaptic and postsynaptic terminals (Chauvet et al., 2003). Delta-catenin has been shown to localize to growth cones, postsynaptic terminals, and dendritic segments (Martinez et al., 2003; Arikkath et al., 2008; Yuan et al., 2015; Baumert et al., 2020). In contrast, the subcellular localization of ARVCF-catenin and p0071-catenin in neurons has not yet been investigated.

p120-catenin and delta-catenin have been found to regulate dendrite morphology in neurons through the cadherin-catenin complex and by modulating small Rho GTPase activity (Elia et al., 2006; Arikkath et al., 2008; Kim et al., 2008; Edbauer et al., 2009; Donta et al., 2022). Depletion of p120-catenin and delta-catenin each result in decreased dendritic length and branching, while increasing delta-catenin expression increased dendritic length and branching in neurons (Elia et al., 2006; Arikkath et al., 2008; Kim et al., 2008; Edbauer et al., 2009; Baumert et al., 2020). To date, fewer studies on ARVCF-catenin and p0071-catenin in neurons have been done but they have each been shown to modulate the cytoskeleton through the cadherin-catenin complex and by modulating small Rho GTPase activity in other cell types (Fang et al., 2004; Keil et al., 2013). While the effects of delta-catenin and p120-catenin on dendrite morphology have been partially characterized, we aimed for the first time to elucidate the roles ARVCF-catenin and p0071-catenin’s effects on dendrite morphogenesis during development, and also to directly compare phenotypic differences with those involving delta-catenin and p120-catenin. Indeed, even with some larger similarities in structure and function of the p120-catenin subfamily members, direct comparisons within the same primary neuronal culture model provide an opportunity to evaluate the relative protein distributions, and importantly, the gross morphologic roles of each subfamily member.

## Results

### p120-subfamily catenins exhibit unique distribution patterns in developing neurons

**FIGURE 2:**
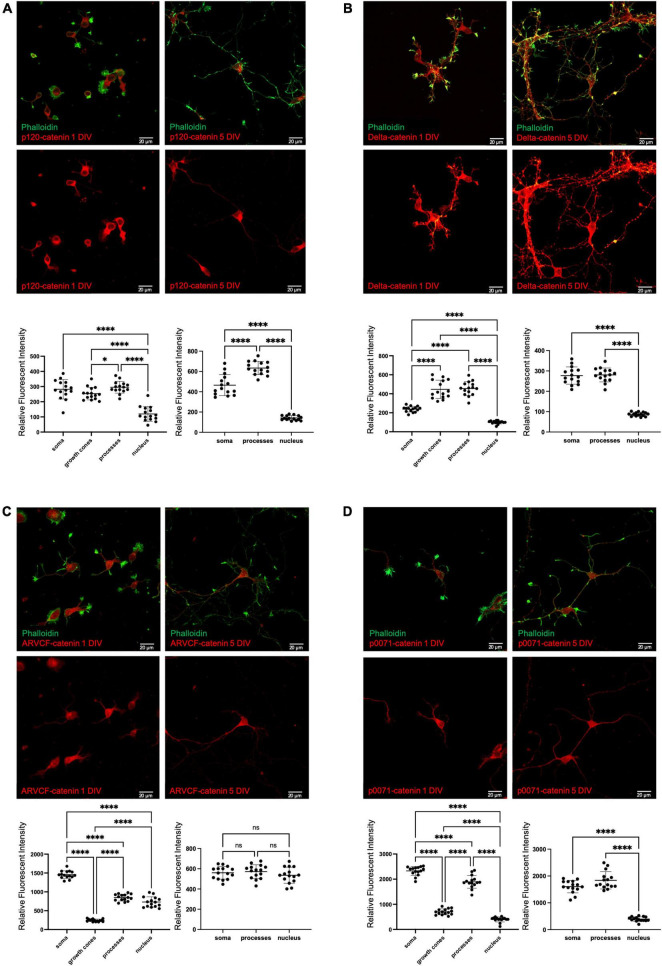
Endogenous localization of p120-subfamily catenins in primary neurons. Endogenous localization of **(A)** p120-catenin; **(B)** delta-catenin; **(C)** ARVCF-catenin; **(D)** p0071-catenin in primary rat hippocampal neurons at 1 DIV and 5 DIV. Relative fluorescent intensity in the soma growth cones, processes, and nucleus is shown below each example image for 1 DIV (left) and 5 DIV (right). Cells were co-stained with Phalloidin and DAPI to help visualize growth cones and nuclei for quantification, but for clarity these stains are not pictured in example images. Statistical significance was determined using a two-way Brown-Forsythe and Welch ANOVA tests with Dunnett’s T3 multiple comparisons *post-hoc* test. Significance was assigned at *P* < 0.05 for *, and *P* < 0.0001 for ****, and ns for non-significant. *N* = 15 neurons from three biological replicates for each catenin at each DIV. Graph values show mean and standard deviation.

Given that there has not yet been a comprehensive comparative study of the p120-subfamily catenins in neurons, we began by examining the cellular localization patterns of p120-catenin, delta-catenin, ARVCF-catenin, and p0071-catenin. While RNA levels of each catenin in the brain are known, our aim was to determine subcellular protein expression. To conduct this study, primary rat hippocampal neurons were fixed at 1 DIV (day *in vitro)* and 5 DIV. The timepoint of 1 DIV was chosen to best visualize growth cones, while 5 DIV was chosen to better visualize dendritic processes. Using general criteria (dendrites, soma, nucleus, growth cones, etc.), antibodies specifically directed against the endogenous catenins were used to visualize and quantify the localization pattern of each p120-subfamily catenin (Figure 2). Since each antibody employed has a different inherent sensitivity to detecting its target catenin protein, we focused upon comparing the relative subcellular distributions within neurons of each catenin protein (not reporting on absolute levels). Each antibody employed was validated through shRNA-mediated knockdown and consequent loss of immunofluorescence staining (Supplementary Figure 1).

At 1 DIV, p120-catenin is present at similar levels in the dendritic processes and soma, followed by the growth cones, and has relatively low presence in the nucleus. At 5 DIV, p120-catenin is most highly present in the processes, followed by the soma, and remains at relatively low levels in the nucleus (Figure 2A), which is consistent with previous studies (Elia et al., 2006). Delta-catenin localizes most to dendritic processes and growth cones, followed by the soma, then the nucleus at 1 DIV (Figure 2B). At 5 DIV, delta-catenin is present at similar higher relative levels in the processes and soma than in the nucleus (Figure 2B). Delta-catenin’s presence in growth cones, dendritic processes, cytoplasm, and cell membrane are consistent with previous findings from us and others (Martinez et al., 2003; Arikkath et al., 2008; Yuan et al., 2015; Baumert et al., 2020). ARVCF-catenin is most present in the soma at 1 DIV, followed by the processes and nucleus, and is least present in the growth cones (Figure 2C). However, at 5 DIV, ARVCF-catenin is about equally distributed throughout the soma, processes, and nucleus (Figure 2C). Finally, at 1 DIV, p0071-catenin is most present in the soma and processes, considerably less present in the growth cones, and even less present in the nucleus (Figure 2D). Similarly, at 5 DIV, p0071-catenin is more present in the soma and processes than the nucleus (Figure 2D).

### p120-subfamily catenins exhibit both shared and distinct effects on dendrite morphology

We sought to determine how altering the expression levels of each p120-subfamily catenin affects dendrite morphology. To do this, we overexpressed versus knocked down each p120-catenin subfamily member in primary rat hippocampal neurons, and we then analyzed the resulting impact upon dendrite morphology. These neurons came from mixed cultures that included all regions in the hippocampus. Neuron-specific expression of each construct was achieved using the hSyn (synapsin 1) promoter so that we only scored neurons (Shevtsova et al., 2005). Furthermore, overexpression constructs contained a GFP reporter that was detached from the rest of the open reading frame *via* a P2A ribosomal skipping sequence. This was done to produce cell fills of GFP of the transfected neurons to visualize their entire morphology while marking the specific neurons expressing the protein of interest. For morphologic analysis, we utilized the Imaris Filament Tracer software, an example of the workflow is shown in Supplementary Figure 2.

**FIGURE 3:**
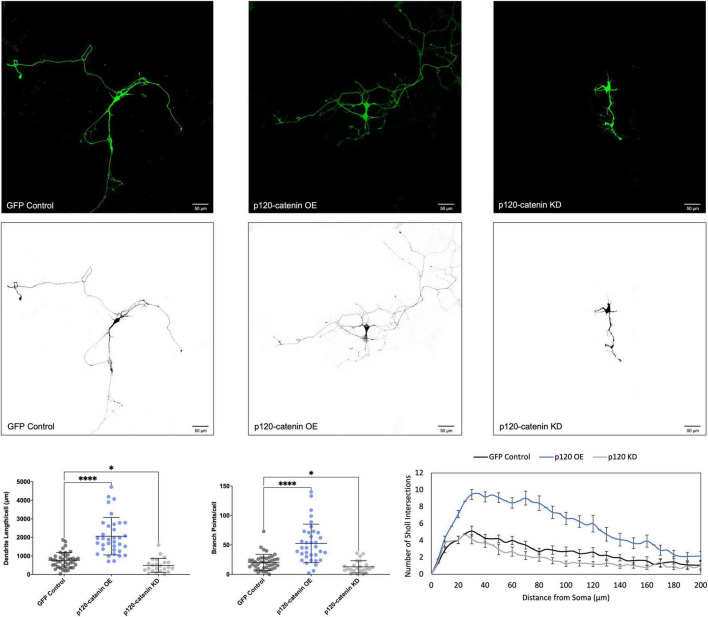
Morphological effects of p120-catenin expression in developing rat primary hippocampal neurons. Example images are shown of neurons that have been transfected with GFP, exogenous p120-catenin, and shRNA to knockdown p120-catenin. Cells were transfected at 3 DIV and analyzed at 7 DIV. These images have been masked to eliminate other cells in the visual field for clarity. Quantification of the average dendrite length per cell and average number of branch points per cell per each condition are shown below on the left, and Sholl analysis for each condition to the right. The number of cells analyzed in each condition were 35 for p120-catenin OE, 23 for p120-catenin KD, and 41 for GFP control. Samples were taken from five biological replicates. Statistical significance was determined using a two-way Brown-Forsythe and Welch ANOVA tests with Dunnett’s T3 multiple comparisons *post-hoc* test. Significance was assigned at *P* < 0.05 for * and *P* < 0.0001 for ****. Chart values show mean and standard deviation.

**FIGURE 4:**
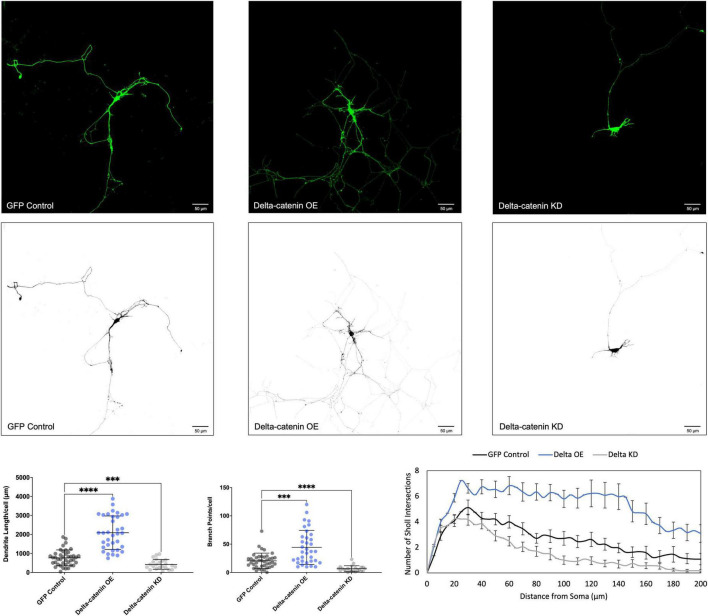
Morphological effects of delta-catenin expression in developing rat primary hippocampal neurons. Example images are shown of neurons that have been transfected with GFP, exogenous delta-catenin, and shRNA to knockdown delta-catenin. Cells were transfected at 3 DIV and analyzed at 7 DIV. These images have been masked to eliminate other cells in the visual field for clarity. Quantification of the average dendrite length per cell and average number of branch points per cell per each condition are shown below on the left, and Sholl analysis for each condition to the right. The number of cells analyzed in each condition were 35 for delta-catenin OE, 23 for delta-catenin KD, and 41 for GFP control. Samples were taken from five biological replicates. Statistical significance was determined using a two-way Brown-Forsythe and Welch ANOVA tests with Dunnett’s T3 multiple comparisons *post-hoc* test. Significance was assigned at *P* < 0.001 for *** and *P* < 0.0001 for ****. Chart values show mean and standard deviation.

**FIGURE 5:**
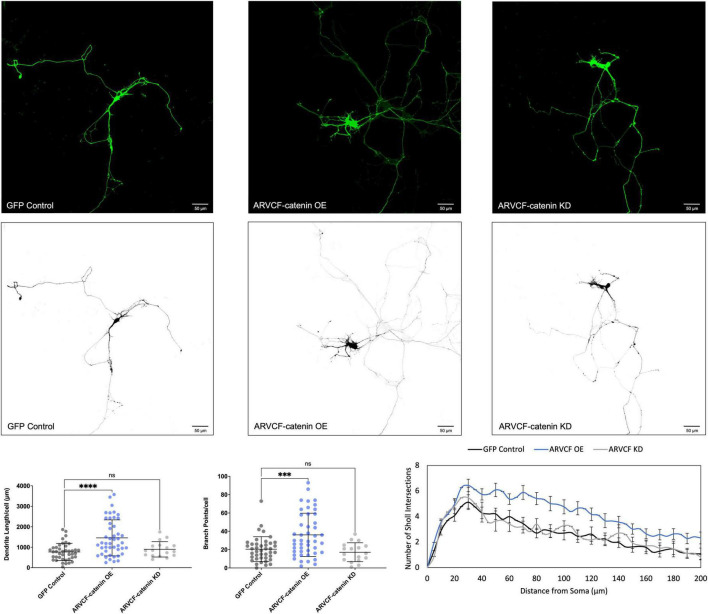
Morphological effects of ARVCF-catenin expression in developing rat primary hippocampal neurons. Example images are shown of neurons that have been transfected with GFP, exogenous ARVCF-catenin, and shRNA to knockdown ARVCF-catenin. Cells were transfected at 3 DIV and analyzed at 7 DIV. These images have been masked to eliminate other cells in the visual field for clarity. Quantification of the average dendrite length per cell and average number of branch points per cell per each condition are shown below on the left, and Sholl analysis for each condition to the right. The number of cells analyzed in each condition were 46 for ARVCF-catenin OE, 17 for ARVCF-catenin KD, and 41 for GFP control. Samples were taken from five biological replicates. Statistical significance was determined using a two-way Brown-Forsythe and Welch ANOVA tests with Dunnett’s T3 multiple comparisons *post-hoc* test. Significance was assigned at *P* < 0.001 for ***, *P* < 0.0001 for ****, and ns for non-significant. Chart values show mean and standard deviation.

**FIGURE 6:**
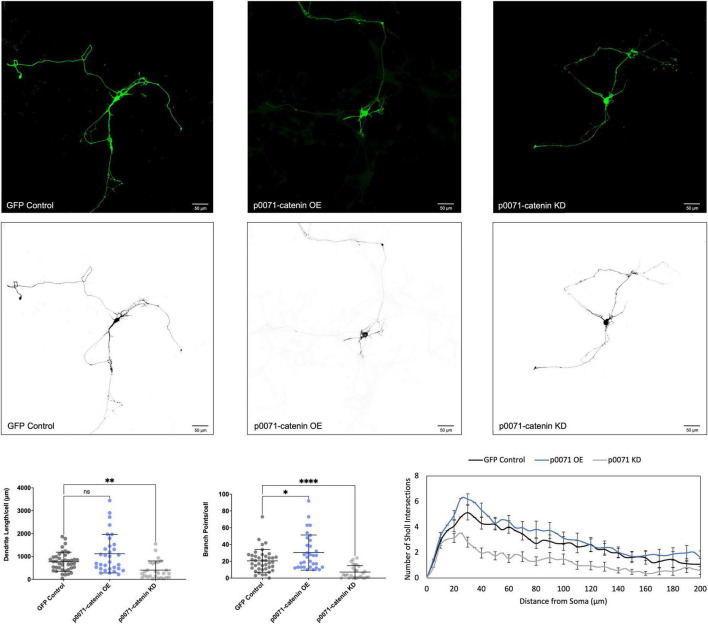
Morphological effects of p0071-catenin expression in developing rat primary hippocampal neurons. Example images are shown of neurons that have been transfected with GFP, exogenous p0071-catenin, and shRNA to knockdown p0071-catenin. Cells were transfected at 3 DIV and analyzed at 7 DIV. These images have been masked to eliminate other cells in the visual field for clarity. Quantification of the average dendrite length per cell and average number of branch points per cell per each condition are shown below on the left, and Sholl analysis for each condition to the right. The number of cells analyzed in each condition were 32 for p0071-catenin OE, 27 for p0071-catenin KD, and 41 for GFP control. Samples were taken from five biological replicates. Statistical significance was determined using a two-way Brown-Forsythe and Welch ANOVA tests with Dunnett’s T3 multiple comparisons *post-hoc* test. Significance was assigned at *P* < 0.05 for *, *P* < 0.01 for **, *P* < 0.0001 for ****, and ns for non-significant. Chart values show mean and standard deviation.

We determined that increasing p120-catenin levels significantly increases dendritic length and branching, while decreasing p120-catenin levels decreases dendritic length and branching (Figure 3). Like p120-catenin, increasing delta-catenin levels increases dendritic length and branching, while decreasing levels decreases dendritic length and branching (Figure 4). Interestingly, we found that increasing the levels of ARVCF-catenin increases dendritic length and branching, but decreasing ARVCF-catenin levels has no effect on dendrite morphology (Figure 5). While p0071-catenin knockdown resulted in decreased dendritic length and branching, we found that overexpressing p0071-catenin increased dendritic branching but did not significantly increase total dendritic length (Figure 6). The effects of each p120-subfamily catenin on dendrite length and branching are shown in Table 1.

**TABLE 1:** Summary of p120-catenin subfamily expression effects on dendrite length and branching in rat primary hippocampal neurons.

	Overexpression		Knockdown	
	Dendrite length	Dendrite branching	Dendrite length	Dendrite branching
p120-catenin	Increase	Increase	Decrease	Decrease
Delta-catenin	Increase	Increase	Decrease	Decrease
ARVCF-catenin	Increase	Increase	No effect	No effect
p0071-catenin	No effect	Increase	Decrease	Decrease

## Discussion

While we observe that each catenin is present in neuronal dendritic growth cones to some degree, a relatively larger proportion of p120-catenin and delta-catenin locate to growth cones compared to ARVCF-catenin and p0071-catenin. As growth cones are sites of neurite outgrowth, p120-catenin and delta-catenin may play a relatively more substantial role in dendritic morphogenesis than ARVCF-catenin and p0071-catenin (Halpain et al., 2009; Raper, 2009). All four catenins are present in dendritic processes, which may suggest that they all play roles in dendrite morphogenesis and/or maintenance. All four p120-subfamily catenins have some degree of localization to the nucleus, which is consistent with studies done in other cell types (McCrea et al., 2015; McCrea and Gottardi, 2016). However, their individual roles in regulating neuronal gene transcription have not been assessed. ARVCF-catenin clearly has the highest proportion of subcellular localization in the nucleus compared to the other three subfamily members. ARVCF-catenin, along with p120-catenin, has been shown to localize highly to the nucleus in non-neuronal cell types as well, but little is known about their potential roles in the nucleus (Daniel and Reynolds, 1999; Mariner et al., 2000; Kelly et al., 2004; Lee et al., 2014). None of the four p120-subfamily catenins have predicted DNA binding domains, but p120-catenin and delta-catenin have been shown to regulate transcription through other effectors, albeit in non-neuronal cell types (Daniel and Reynolds, 1999; Kelly et al., 2004; Rodova et al., 2004; Park et al., 2006; Hosking et al., 2007; Kim et al., 2008; Hong et al., 2010; Gu et al., 2011; Lee et al., 2014; Nopparat et al., 2015; Kulikov et al., 2016). Thus, it is currently unknown if the presence of any p120-subfamily catenin in the nucleus may be contributory to dendritic arborization.

The effects of p120-catenin knockdown on dendrite morphology align with previous work, which showed that p120-catenin knockdown decreases the dendritic arbor in CA1 primary hippocampal neurons from p120-catenin knockout mice *in vitro* (Elia et al., 2006). Our results showing that p120-catenin overexpression increases dendritic arborization are complimentary, as the impacts are the converse effect of p120-catenin knockdown. The neuronal phenotype corresponding to p120-catenin knockdown is thought to be primarily caused by p120-catenin’s interactions with cadherins and the small Rho GTPases RhoA and Rac1 (Elia et al., 2006). p120-catenin has been shown to inhibit RhoA and activate Rac1 in multiple cell types, including neurons (Anastasiadis et al., 2000; Noren et al., 2000; Grosheva et al., 2001; Elia et al., 2006). RhoA inhibition and Rac1 activation were established to increase dendritic arborization (Nakayama et al., 2000; Tashiro et al., 2000; Tashiro and Yuste, 2004; Guiler et al., 2021; Donta et al., 2022). Thus, our results are consistent with the hypothesis that p120-catenin expression increases dendritic arborization by inhibiting RhoA and activating Rac1, with the converse occurring upon p120-catenin depletion. Based on these depletion studies, it does not appear that p120-catenin depletion is compensated for by any other p120-subfamily catenin.

Morphological changes due to delta-catenin overexpression and knockdown are consistent with previous studies. There are multiple plausible mechanisms of how delta-catenin influences the dendritic arbor (Donta et al., 2022). Delta-catenin has been shown to inhibit RhoA and activate Rac1 (Martinez et al., 2003; Abu-Elneel et al., 2008; Kim et al., 2008; Gu et al., 2009; Gilbert and Man, 2016). Furthermore, delta-catenin is also thought to modulate dendrite morphology through its PDZ-binding motif, which p120-catenin lacks (Yuan et al., 2015). Interactions of delta-catenin’s PDZ-binding motif have also been shown to be phospho-dependent in certain cases, where the phosphorylation state of delta-catenin’s PDZ-binding motif dictates whether delta-catenin promotes dendritic length versus elongation (Baumert et al., 2020). Finally, delta-catenin was suggested to regulate dendrite morphogenesis by interactions with effectors like cortactin *via* delta-catenin’s C-terminal region (Martinez et al., 2003; Toyo-oka et al., 2014; Lee et al., 2015). While our results indicating that delta-catenin expression levels have a notable impact upon dendritic arborization are consistent with previous work, additional studies will be needed to reveal the exact mechanism(s) of action. Analogous to the morphological effects evident following the depletion of p120-catenin, those that relate to the knockdown of delta-catenin do not appear to be compensated for by delta’s remaining p120 sub-family members.

Although investigations of ARVCF-catenin in neurons are limited–particularly concerning its influence on dendrite morphology–studies in other cell types have characterized some of its roles in modulating the cytoskeleton. In *X. laevis*, ARVCF-catenin was shown to inhibit RhoA and activate Rac1 in a similar manner to p120-catenin (Fang et al., 2004; Valls et al., 2012). If ARVCF-catenin has the same effect on RhoA and Rac1 in neurons, this would lead to increased arborization upon overexpression, which is consistent with our results (Govek et al., 2005; Stankiewicz and Linseman, 2014). Studies in *X. laevis* and NIH 3T3 cells have also shown that p120-catenin and ARVCF-catenin can rescue each other’s depletions, with partial functional redundancy being a potential explanation for why ARVCF-catenin knockdown in neurons does not result in an altered phenotype (Mariner et al., 2000; Fang et al., 2004). p120-catenin is present in relatively higher protein levels compared to ARVCF-catenin in mammalian brain tissue (Mariner et al., 2000). In our current context of hippocampal neurons, this suggests the possibility that endogenous p120-catenin may largely rescue any gross morphological impact upon ARVCF-catenin depletion, but not the other way around. Furthermore, ARVCF-catenin contains a PDZ-binding motif as does delta-catenin so it perhaps share additionally interactions and thus functionality with delta-catenin and PDZ domain-containing proteins in regulating dendrite morphogenesis, though this has yet to be tested in neurons.

Studies centered on p0071-catenin in neurons are also limited, but our results are consistent with one study that found that p0071-catenin overexpression increased dendritic branching (Nolze et al., 2013). In non-neuronal tissues, p0071-catenin activates RhoA, which differs from p120-catenin, delta-catenin, and ARVCF-catenin (Wolf et al., 2006; Keil et al., 2009, 2013). As interactions with small Rho GTPases are thought to be a major pathway for how p120-subfamily catenins modulate the cytoskeleton, we would expect p0071-catenin to have the opposite morphological effects as the other three catenins. However, there are a few possibilities as to why we did not see such results. p0071-catenin’s interactions with small Rho GTPases could be tissue- and cell-type dependent, so studies showing that p0071-catenin activates RhoA in non-neuronal cell types may not hold true in neurons. Furthermore, as seen for delta-catenin, p0071-catenin may have multiple mechanisms of modulating the cytoskeleton (Elia et al., 2006; Kim et al., 2008). There may be an interplay between p0071-catenin’s interactions with small Rho GTPases and its functional association with other binding partners. This may also explain why p0071-catenin expression increases the number of branch points, but not the total dendritic length of neurons as is seen with p120-catenin, delta-catenin, and ARVCF-catenin. p0071-catenin depletion does not appear to be compensated for by any other p120-subfamily catenin.

Altering p120-subfamily expression in neurons increased sample variance within conditions (i.e., sample variance was relatively higher when altering p120-subfamily expression compared to the GFP control). This is likely due to the inclusion of multiple types of neurons in the cultures. These primary cultures originating from the rat hippocampus contained neurons from the CA1, CA2, CA3 regions, the dentate gyrus, and the subiculum complex (Lee et al., 2017). Therefore, it is possible that altering the expression of these catenins produces varied effects upon the distinct types of neurons present. Further, while our study focuses on the gross morphologies of dendrites, axons were not eliminated from analysis. This was due to the required high-density plating of neuronal cultures which generates extensive overlap of processes, raising difficulties in our ability to distinguish dendrites from axons using stains recognizing MAP2 and Tau. That considered, due to the multiplicity of dendrites per neuron (relative to the presence of a single axon), the predominance of observed morphological changes seen after altering expression of p120-subfamily members derives from effects upon dendrites.

## Conclusion

Overall, the relative localization patterns of the p120-catenin subfamily are consistent with the possibility that they each play roles in dendritic arborization and maintenance, with p120-catenin and delta-catenin potentially playing a relatively stronger role in dendrite morphogenesis than ARVCF-catenin and p0071-catenin based on their relative abundance in growth cones. Furthermore, our morphologic analysis suggests that these catenins contain some shared and some distinct effects on dendrite shaping during neuronal development. The effects of the p120-subfamily catenins on dendrite morphology are likely due in part to their interactions with small Rho GTPases. However, further studies are needed to determine if the effects of each catenin are primarily caused by modulating small Rho GTPase activity, other effector molecules, or a combination of both. Finally, as abnormal dendrite morphology is seen in multiple neurological disorders like Alzheimer’s disease, schizophrenia, and autism spectrum disorder, determining the mechanisms of how each catenin modulates dendrite morphology may help point to new therapeutic approaches or considerations for these diseases. Interestingly, p120-subfamily catenins, most prominently delta, have associations with pathological states involving neurons and brain functions (Xie et al., 2005; Nolze et al., 2013; Donta et al., 2022).

## Materials and methods

### Cell culture and transfections

Primary hippocampal neurons were isolated from rat embryos at E18 as previously described (Baumert et al., 2020). All protocols involving vertebrate animals were approved by the Institutional Animal Care and Use Committee prior to initiating the studies. Once isolated, the hippocampal neurons were plated in 24-well tissue-culture plates with coverslips coated with 100 μg/mL poly-D-lysine at a density of 2 × 10^5^ cells per well. The cultures were maintained in Neurobasal Medium supplemented with B-27, GlutaMAX, and penicillin-streptomycin. Transfections in neurons were done at 3 DIV using Lipofectamine 2000 according to the manufacturer’s protocol.

### Immunocytochemistry

Neurons were fixed with 4% paraformaldehyde for 10 min at room temperature, incubated for 5 min in PBS supplemented with 100 mM glycine, washed with PBS for 30 min, then permeabilized with 0.5% Triton X-100 for 10 min at room temperature. The neurons were then blocked with PBS containing 1% bovine serum albumin overnight at 4°C. After blocking, the neurons were incubated with primary antibodies in blocking solution overnight at 4°C. The neurons were washed with PBS 3 times, then incubated with secondary antibodies for 1 h at room temperature. Following a second washing step of 3 washes at 5 min each, coverslips were mounted onto slides with 2 μL of VECTASHIELD Antifade Mounting Medium.

### Confocal microscopy

Neurons were imaged using a Nikon T2i Inverted Confocal Microscope with a 20× Plan Achromat 0.75 NA lens for morphology studies and an Apo-Plan 60 × 1.4 NA oil objective for endogenous localization studies. Images were captured with a Nikon A1-DUG GaAsP hybrid four-channel multi-detector and Nikon NIS-Elements software. All z-series images were acquired at a pixel size of 100 nm and a step size of 1.1 μm at 20× and 0.2 μm at 60×. Excitation was done with a 488 nm laser line and emission was collected with a BP 525/50 m filter. The pinhole was set at 1 airy unit for all images. For imaging endogenous localization of catenins, the laser intensity, offset, and high voltage settings on the microscope were kept consistent between images of the same condition. All images were acquired below the threshold for pixel saturation. For morphological analysis of images acquired at 20×, montages of at least 5 × 5 tiles were captured and stitched together to visualize larger fields of view.

### Antibodies

p120-catenin antibody (Invitrogen, Cat. #33-9600) was used at 1:250 dilution. Delta-catenin antibody (BD Technologies, Cat. #61137) was used at 1:250 dilution. ARVCF-catenin antibody (Santa Cruz Biotechnology, Cat. #sc-23874) was used at 1:100 dilution. p0071-catenin antibody (Invitrogen, Cat. #PA5-101752) was used at 1:250 dilution. For secondary antibodies, Alexa Fluor 488 (Cat. #A-11001, #A-11008) was used at 1:1,000 dilution, Alexa Fluor 555 (Cat. #A32732) was used at 1:1,000 dilution, and Alexa Fluor 568 (Cat. #A-11004) was used at 1:1,000 dilution.

### Plasmids

All cDNA transfections for overexpression analyses utilized an AAV vector, an eGFP reporter, an hSyn promoter, and a P2A ribosomal skipping sequence to separate the exogenous catenin from the eGFP reporter to visualize the neurons’ full morphology. All shRNA-mediated knockdowns utilized a pSUPER vector with an eGFP reporter. The GFP controls contained only the vectors and eGFP. Overexpression plasmids were transfected with Lipofectamine 2000 according to the manufacturer’s protocol at 0.5 μg/well, and knockdown plasmids were transfected at 0.25 μg/well. The human forms of each catenin were used for exogenous expression plasmids and are as follows: (CTNND1, Gene ID:1500; CTNND2, Gene ID: 1501; ARVCF, Gene ID:421; PKP4, Gene ID:850). The sequences for the shRNA targeting each catenin are as follows: p120-catenin, 5′-CGATATTGGTAGGATTGTT-3′; delta-catenin, 5′-TAGAAGTCGACATAGTTGC-3′; ARVCF-catenin, 5′-CGAGTTAGCTATTTACTGA-3′; p0071-catenin, 5′-GGACTGTTCATGACATGGA-3′.

### Image analysis

After capturing confocal images in the manner described above, several analyses were performed on the neurons. Endogenous localization was quantified using ImageJ by measuring the relative average fluorescent intensity of each catenin compared to itself in the nucleus, soma, processes, and growth cones. These measurements were obtained by going through each slice of the image, creating regions of interest for the four different subcellular regions, and using ImageJ to measure the average fluorescent intensity of each region. This process was repeated until all slices of the image were measured. The values presented for each cell are the average value for each slice to account for differences in thickness. Cells were co-stained with DAPI and phalloidin to determine subcellular regions. Measurements were averaged from 15 neurons for each condition from three separate cultures for endogenous localization studies. Fluorescent intensity is to be compared between subcellular localizations for the same catenin but is not to be compared between different catenins. For morphological analysis, cells were transfected at 3 DIV and analyzed at 7 DIV. Imaris Filament Tracer was used to create a skeleton of each neuron. From this tracing, the software automatically measures dendrite length, dendrite branching, and performs Sholl analysis. The radii interval for sholl analysis was set at 5 μm. Laser intensity and detector settings were kept the same between samples while collecting images, and all threshold settings were kept the same between samples when running the Imaris Filament Tracer software. Processes shorter than 5 μm were excluded from the analysis. Sample sizes were between 17 and 46 neurons from 5 biological replicates for each condition.

### Antibody and shRNA validation

To validate antibodies and shRNA knockdowns, primary rat hippocampal neurons were transfected with shRNA to knockdown each p120-subfamily catenin at 3 DIV, then were fixed at 7 DIV. Samples were incubated with antibodies targeting the respective endogenous catenin, then imaged. Fiji was used to measure the fluorescent intensity of endogenous catenin in cells that were transfected with shRNA and were compared against cells that were transfected with GFP as a control.

### Statistical analysis

Data were analyzed using GraphPad Prism. Statistical significance for all endogenous localization study and morphological analysis comparisons were determined using a two-way Brown-Forsythe and Welch ANOVA tests with Dunnett’s T3 multiple comparisons *post-hoc* test. Statistical significance for antibody and shRNA knockdown validation was determined using unpaired *t*-tests with Welch’s correction. Significance was assigned at *P* < 0.05 for *, *P* < 0.01 for ^**^, *P* < 0.001 for ^***^, and *P* < 0.0001 for ^****^.

## Data availability statement

The original contributions presented in this study are included in the article/Supplementary material, further inquiries can be directed to the corresponding authors.

## Ethics statement

The animal study was reviewed and approved by the Institutional Animal Care and Use Committee.

## Author contributions

MD: concept of study, research design, data collection and analysis, and manuscript preparation. YS: data collection and protein modeling. CD: hippocampal culture preparation and experimentation. AP-H: experimentation, imaging, and analysis. MW: concept of study, research design, and manuscript review and editing. PM: concept of study, research design, manuscript preparation, and review and editing. All authors contributed to the article and approved the submitted version.

## Supplementary material

Supplementary Figure 1 p120-catenin subfamily antibody validation *via* shRNA-mediated knockdown. Rat primary hippocampal neurons were transfected at 3 DIV with shRNA plasmids designed to knockdown p120-catenin, delta-catenin, ARVCF-catenin, and p0071-catenin, then fixed at 7 DIV. Green cells in the images are neurons that have been transfected with shRNA vectors corresponding to the labeled catenin on the image, and magenta cells depict the respective endogenous p120-subfamily catenin antibody. For each catenin, the image on the left includes the neuron(s) that has been transfected with shRNA knockdown, and the image on the right shows the neurons without the transfected neuron(s). Neurons that have been transfected with shRNA knockdown have significantly lower staining intensity, indicating that the shRNA knockdown decreased the amount of catenin present. Dots in each bar graph indicate number of cells analyzed for each condition and average and standard deviations are indicated. ^****^ represent *P*-values < 0.0001.

Supplementary Figure 2 The Imaris Filament Tracer software used to create a tracing of a transfected neuron. The image on the left is a 7 DIV rat primary hippocampal neuron that has been transfected with exogenous ARVCF-catenin with an eGFP reporter (magenta). The MAP2 neuronal marker (cyan) was used to depict neuronal dendrites, and DAPI (yellow) was used to visualize the nucleus. The image on the right is the skeleton of the transfected neuron produced by the Imaris Filament Tracer software. The tracings are used to quantify dendrite length and branching, as well as to perform Scholl analysis.
