# Supplementary figures and images for: p120-catenin subfamily members have distinct as well as shared effects on dendrite morphology during neuron development in vitro
Source: Front Cell Neurosci. 2023 Apr 4;17:1151249. doi: 10.3389/fncel.2023.1151249 (PMC10112520; doi:10.3389/fncel.2023.1151249)

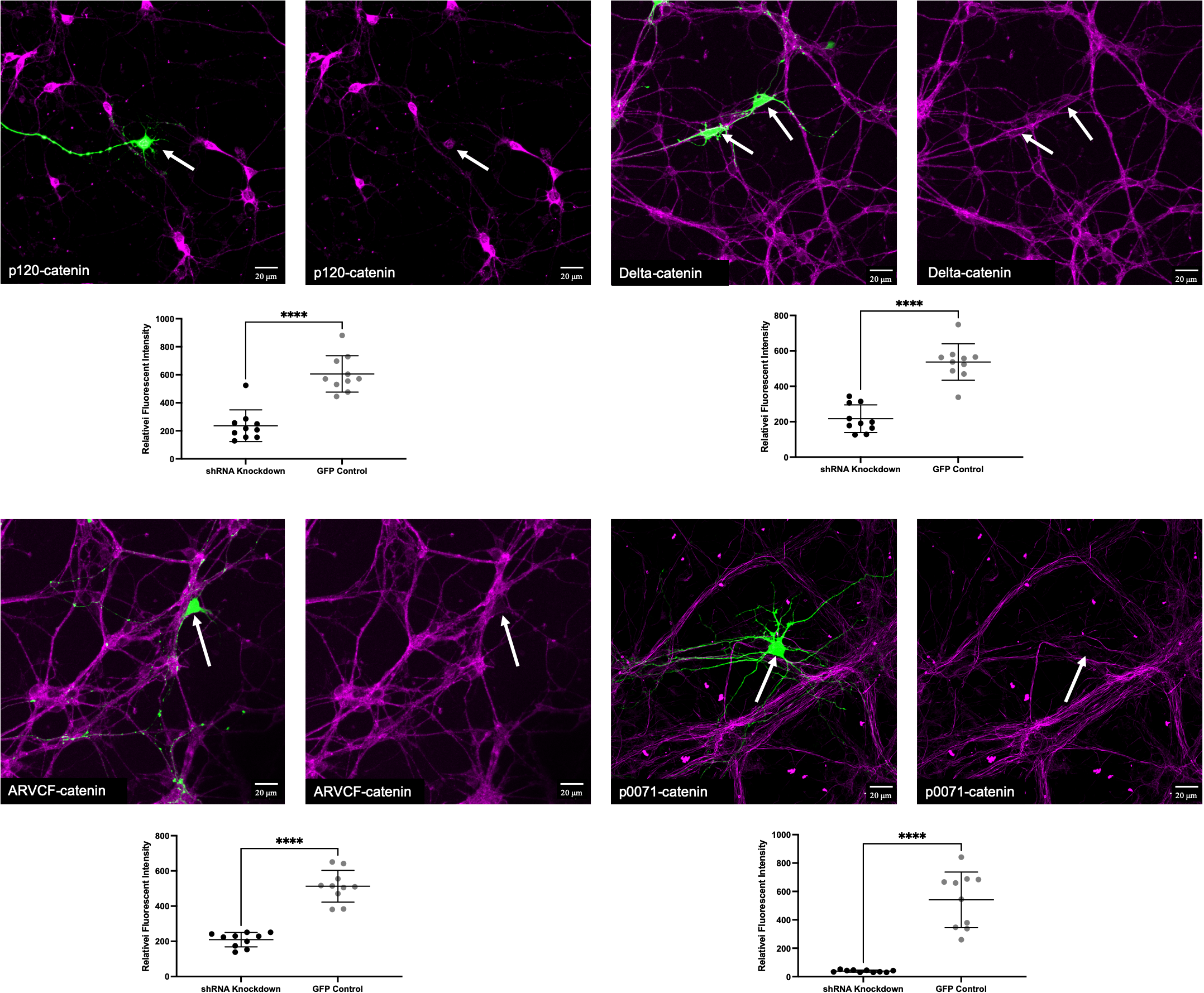

Supplement: Supplementary Figure 1 — p120-catenin subfamily antibody validation via shRNA-mediated knockdown. Rat primary hippocampal neurons were transfected at 3 DIV with shRNA plasmids designed to knockdown p120-catenin, delta-catenin, ARVCF-catenin, and p0071-catenin, then fixed at 7 DIV. Green cells in the images are neurons that have been transfected with shRNA vectors corresponding to the labeled catenin on the image, and magenta cells depict the respective endogenous p120-subfamily catenin antibody. For each catenin, the image on the left includes the neuron(s) that has been transfected with shRNA knockdown, and the image on the right shows the neurons without the transfected neuron(s). Neurons that have been transfected with shRNA knockdown have significantly lower staining intensity, indicating that the shRNA knockdown decreased the amount of catenin present. Dots in each bar graph indicate number of cells analyzed for each condition and average and standard deviations are indicated. **** represent P-values < 0.0001. [file Image_1.tiff]

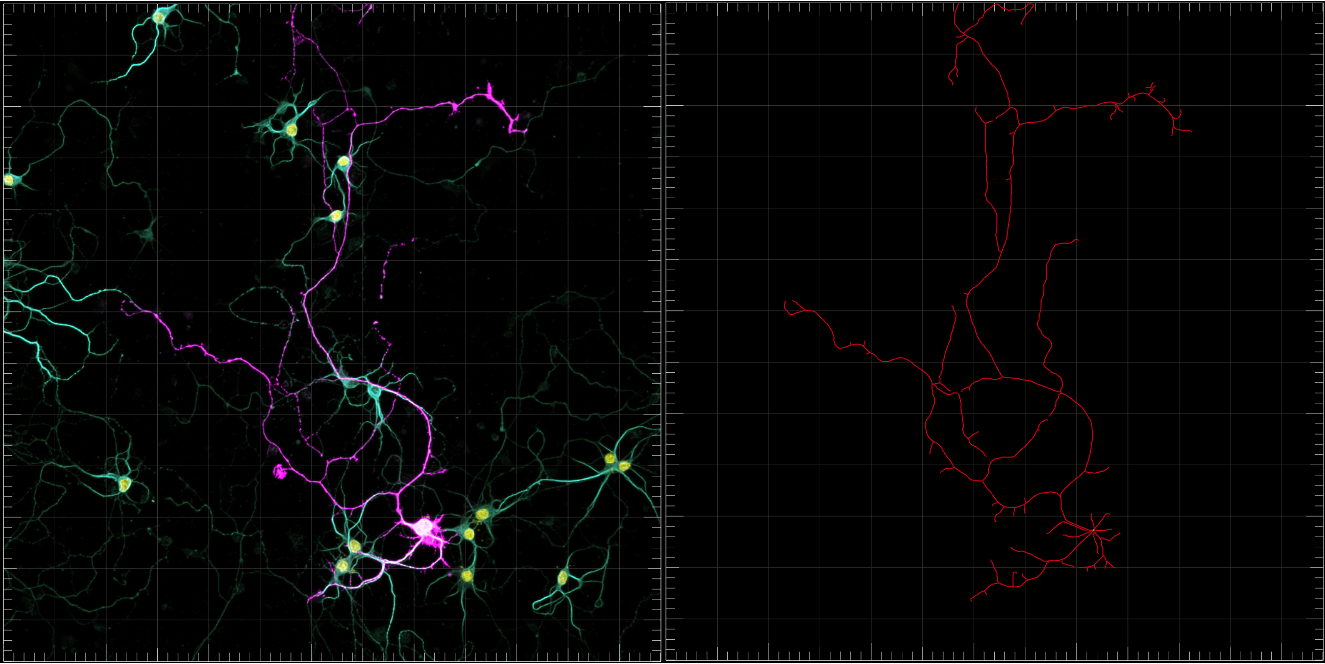

Supplement: Supplementary Figure 2 — The Imaris Filament Tracer software used to create a tracing of a transfected neuron. The image on the left is a 7 DIV rat primary hippocampal neuron that has been transfected with exogenous ARVCF-catenin with an eGFP reporter (magenta). The MAP2 neuronal marker (cyan) was used to depict neuronal dendrites, and DAPI (yellow) was used to visualize the nucleus. The image on the right is the skeleton of the transfected neuron produced by the Imaris Filament Tracer software. The tracings are used to quantify dendrite length and branching, as well as to perform Scholl analysis. [file Image_2.tiff]
